# Doubtful outcome of the validation of the Rome II questionnaire: validation of a symptom based diagnostic tool

**DOI:** 10.1186/1477-7525-7-106

**Published:** 2009-12-29

**Authors:** Herdis KM Molinder, Lars Kjellström, Henry BO Nylin, Lars E Agréus

**Affiliations:** 1 Centre for Family and Community Medicine, Karolinska Institutet, Nobels Allé 12, 141 52 Huddinge, Sweden; 2 Department of Medicine, Huddinge, Karolinska Institutet, Stockholm, Sweden; 3 Centre for Family and Community Medicine. Karolinska Institutet, Stockholm, Sweden

## Abstract

**Background:**

Questionnaires are used in research and clinical practice. For gastrointestinal complaints the Rome II questionnaire is internationally known but not validated. The aim of this study was to validate a printed and a computerized version of Rome II, translated into Swedish. Results from various analyses are reported.

**Methods:**

Volunteers from a population based colonoscopy study were included (n = 1011), together with patients seeking general practice (n = 45) and patients visiting a gastrointestinal specialists' clinic (n = 67). The questionnaire consists of 38 questions concerning gastrointestinal symptoms and complaints. Diagnoses are made after a special code. Our validation included analyses of the translation, feasibility, predictability, reproducibility and reliability. Kappa values and overall agreement were measured. The factor structures were confirmed using a principal component analysis and Cronbach's alpha was used to test the internal consistency.

**Results and Discussion:**

Translation and back translation showed good agreement. The questionnaire was easy to understand and use. The reproducibility test showed kappa values of 0.60 for GERS, 0.52 for FD, and 0.47 for IBS. Kappa values and overall agreement for the predictability when the diagnoses by the questionnaire were compared to the diagnoses by the clinician were 0.26 and 90% for GERS, 0.18 and 85% for FD, and 0.49 and 86% for IBS. Corresponding figures for the agreement between the printed and the digital version were 0.50 and 92% for GERS, 0.64 and 95% for FD, and 0.76 and 95% for IBS. Cronbach's alpha coefficient for GERS was 0.75 with a span per item of 0.71 to 0.76. For FD the figures were 0.68 and 0.54 to 0.70 and for IBS 0.61 and 0.56 to 0.66. The Rome II questionnaire has never been thoroughly validated before even if diagnoses made by the Rome criteria have been compared to diagnoses made in clinical practice.

**Conclusion:**

The accuracy of the Swedish version of the Rome II is of doubtful value for clinical practice and research. The results for reproducibility and reliability were acceptable but the outcome of the predictability test was poor with IBS as an exception. The agreement between the digital and the paper questionnaire was good.

## Introduction

Gastrointestinal complaints cause about 5% of all the annual visits in primary health care and about 50% of these are referred to gastroenterologists [1-4]. A majority of the symptoms is caused by functional gastrointestinal disorders (FGID), often linked to somatic symptoms from other parts of the body. FGIDs might also affect mental health and cause an impact on the patient's quality of life [5,6]. However, FGID is still an exclusion diagnosis, that is, a diagnosis made after organic causes have been reasonably excluded [7]. In epidemiological research FGIDs are diagnosed only on the basis of symptoms, presuming that the proportion of an organic explanation for their complaints is low. This has been shown to be reasonable in epidemiological endoscopy studies [8-10].

At two consecutive meetings in Rome the European Congress on Gastrointestinal Diseases reached consensus about diagnostic criteria for functional gastrointestinal disorders. In 1996, a committee provided a questionnaire: the Rome II Modular Questionnaire, with 38 questions and alternative answers, describing the frequency of recorded symptoms (Additional file 1). The questionnaire includes questions about clusters of symptoms from six organs: the oesophagus, stomach, bowel, abdomen, biliary tract, and rectum and codes for defining various gastrointestinal diagnoses on the basis of the answers to the questionnaire.

Symptom questionnaires are regularly used in research and also, but to a lesser extend, in clinical practice. In clinical and population-based studies as well as in clinical trials questionnaires are useful tools for obtaining broad information of the frequency of certain symptoms, and for clustering of symptoms into domains. In clinical practice a questionnaire may help the doctor to confirm a diagnosis in a structured way.

Computerized versions of questionnaires tend to be more commonly used, especially in research, but to our knowledge no effort has been made to compare the outcome of computerized tools to printed ones. It has been taken for granted that the results will be the same. However, it is always possible to change an answer on a printed questionnaire and also compare various questions in advance, which can lead to nuanced answers. Computerized versions on the other hand lack overviews and have a compulsory step-by-step function. Thus, the results of the printed questionnaire may be different from the computerized one. We therefore compared the outcome of the two versions.

Most questionnaires are developed in English and intended for use in English-speaking countries. Non-English speaking countries can either create their own questionnaires or translate well-known material into their own language. The first option is time-consuming and makes it difficult to compare results internationally. Thus, translating existing tools seems more efficient. However, a mere translation is unlikely to be successful because of language and cultural differences, and every translation must therefore be validated using various criteria [11]. The value of each word, issue and domain must be analysed in relation to its application in the new medical and cultural surroundings. A confirmation of reliability and validity of symptom-based measures is essential. A reliable instrument should also assess the symptoms being most problematic or of most concern, and target the subjects that are not affected by the symptoms in the questionnaire.

Functional gastrointestinal symptoms are commonly divided into three main groups: gastro-oesophageal reflux symptoms (GERS, or functional heartburn (FH)), functional dyspepsia (FD) and irritable bowel syndrome (IBS). Differing definitions of these subgroups make it difficult to compare figures of frequency of symptoms in each subgroup; symptoms also often overlap and change over time [12]. International epidemiological studies show on average a prevalence of FH/GERS of 25%, of FD also 25% and of IBS 12% in the population [13]. However, only a fraction of people with functional gastrointestinal symptoms seeks medical advice. Those who do so, suffer not only from symptoms, but at least to some extent also from fears and worries forming their health care seeking behaviour [14].

Knowing the risk of such bias, an unselected population is preferable for validation of a symptom questionnaire, especially for instruments aimed to be used in both epidemiological studies and for comparison with clinical settings at different levels (primary, secondary or tertiary).

## Aim

The aim of this study was to explore the validity of a Swedish version of the Rome II Patient Modified Formula questionnaire (in this paper called Rome II) with special focus on IBS and to compare the outcome of the printed version to the computerized one.

## Materials and methods

### The Rome II questionnaire

The Rome II Modular Questionnaire: Respondent Form (Additional File 1) consists of 38 questions concerning not only symptoms but also the frequency and severity of the symptoms. The symptoms are presented per organ in supposed functional diagnostic groups. Symptoms are described in sentences that begin, "In the last 3 months, did you often have...." and the choice is "no or rarely" or "yes". "Often" is defined as the presence of symptoms for at least one day per week during three weeks for the past three months. Some of the questions ask for more detailed information about stools or pain and discomfort and also the possible connection between the timing of symptoms and bowel habit disturbances.

The diagnostic terms used in Rome II is: Functional heartburn (FH), Functional dyspepsia (FD) and Irritable bowel syndrome (IBS). The term "functional" means that organic causes of the symptoms are excluded. Organic causes can be excluded only if endoscopy and further work up has been performed. When the questionnaire is used in epidemiologic research, however, such investigations are often deemed unnecessary because of the presumed low prevalence of organic causes in people with gastrointestinal symptoms [8-10]. This is, however, valid only for FD and IBS while persons with GERS to a considerable extend have an organic cause as an explanation [9,15]. Therefore FH is actually an incorrect term to be used in upper gastrointestinal epidemiological research where the subjects are uninvestigated, and thus GERS is more relevant. With this in mind, we will use the term FH/GERS where we refer to the Rome II consensus document, but GERS elsewhere.

Two technical versions of the questionnaire were used: the printed questionnaire (paper version), which was the main object for our validation, and a computerized version.

The English and the Swedish versions of the questionnaire are included as Additional Files 1 and 2.

### The codes for diagnoses

The codes for the diagnoses FH/GERS, FD and IBS demand an answer "yes" to a key question, followed by "yes" or "no" to supporting questions or questions intended to rule out organic causes [7].

Responders could receive more than one diagnosis with the exception of FH/GERS and FD simultaneously. A key question (#8) for FH/GERS and FD must be answered with yes or no.

### Study population groups

Four study populations participated in the study.

A. The main study group consisted of a randomly elected subset (n = 125) from an ongoing population based colonoscopy study in healthy individuals (the Popcol study, n = 1101) [10], who filled in both the printed questionnaire and a digital version of Rome II.

B. Randomly selected patients, seeking medical advice for any disorder in a general practice (n = 45).

C. Patients, who participated in the Popcol study, and visited the gastrointestinal specialists' (GI) clinic on selected days (n = 67).

D All participants in the Popcol study who were eligible for analyze (n = 1101).

### Validation processes

Standard psychometric practices [16] were used to establish the validity of the Swedish translation of the Rome II modular questionnaire.

### Translation

Adequate translation into Swedish was undertaken in several steps following standard international principles.

1. A team of medically educated individuals, whose native language was Swedish translated the questionnaire from English into Swedish

2. A board, consisting of doctors and nurses from various kind of expertise discussed and changed words in the translation.

3. A group of lay readers reviewed the questionnaire, judging the concept.

4. A Swedish-speaking physician whose native language was English translated the corrected text back to English.

5. The team of medically educated individuals compared the two English texts and approved the final version.

### Feasibility

To investigate the degree to which the responders were confident with the questionnaire, randomly selected responders, n = 41 (22 from group B and 19 from group C) answered the following questions anonymously:

1. Was the questionnaire easy to fill in?

2. Were the questions easy to understand?

3. Did the wordings of the questions describe your symptoms correctly?

4. Were descriptions of any symptom missing from the questionnaire?

5. How long did it take to fill in the questionnaire?

### Reproducibility

To determine if the questionnaire consistently resulted in the same diagnoses when given to a patient on repeated occasions, a test-retest procedure was performed by 102 randomly selected participants: 26 from group A, 45 from group B and 31 from group C. All were asked to fill in the questionnaire on two separate occasions with not more than a week's interval. On the first occasion, they were not informed that they would be asked to complete the questionnaire a second time. A new questionnaire was mailed to all respondents along with an explanatory letter, asking them to repeat the procedure. All but one agreed to do so. The results were calculated as kappa values, and the outcome was interpreted as: 0-0.2 poor, 0.2-0.4 fair, 0.4-0.6 moderate, 0.6-0.8 substantial, and 0.8-1.0 almost perfect agreement [17,18].

### Predictability

The ability of the questionnaire to give an accurate diagnosis was analysed by comparing diagnoses from Rome II, both in the digital (n = 1101) and the paper version (n = 125) with the diagnoses made at a clinical investigation by a specialist in gastroenterology, blinded to the results of the filled in questionnaire. Kappa values and overall agreement were measured.

The clinical diagnoses were made after common clinical practice, normally used at the specialists' clinic and before any laboratory or endoscopic tests. Five specialists were involved in the diagnostic process and consensus meetings were performed before and twice annually during the study. These meetings were guided by a researcher familiar with the Rome II terminology regarding FH/GERS, FD and IBS.

Kappa values and overall agreement were measured.

### Reliability

Principal Component Analysis (PCA) was performed to establish the value of various symptoms in the chosen diagnoses by analyzing selected questions from the complete questionnaire. All completed paper questionnaires from group A and B and C were used (n = 237). Only questions confirming symptoms were included in the analysis; questions on frequency or consequences of symptoms, or questions negating symptoms were left out. We analysed a "short" version which included only the questions relevant for (and used in the Rome II algorithms) for the diagnoses FH/GERS, FD, and IBS (Table 1) and the "full" version which included all symptom (but not non-symptom) questions (Table 2). The factor structures were confirmed using a PCA with varimax rotation [17].

**Table 1:** The rotated (short version) PCA of only the symptoms used for the diagnoses FH, FD, and IBS in the Rome II Modular Questionnaire with four descriptively labelled factors in descending eigenvalues.

Eigenvalue	6.38	3.51	2.09	1.81
Factor label	IBS/diarrhoea	GERS	Dyspepsia/heartburn	IBS/Constipation
Change in stool frequency	**0,77**	-0,10	-0,18	0,13
Change in stool consistency	**0,77**	-0,03	-0,20	0,17
Lower abdominal pain or discomfort (PoD)	**0,66**	-0,06	-0,46	0,22
Loose stools	**0,64**	0,11	0,15	0,19
> three bowel movements a day	**0,59**	0,24	0,01	0,00
PoD diminishes after bowel movements	**0,58**	-0,24	-0,24	0,23
Loose stools 3/4 of times	**0,57**	0,34	0,14	0,08
Urgency	**0,53**	0,11	-0,12	0,05
Nausea or vomiting	0,03	**0,71**	0,01	0,13
Food regurgitates	0,12	**0,70**	-0,16	-0,04
Chestpain	-0,03	**0,68**	-0,20	0,21
Regurgitation stops when food turns acid	0,10	**0,65**	-0,10	0,01
Difficult swallowing	0,11	**0,60**	-0,23	0,02
Frequent episodes of vomiting	-0,11	**0,60**	0,15	0,33
Difficult or painful swallowing	0,05	**0,49**	-0,27	-0,07
A lump in your throat	0,13	**0,42**	-0,23	-0,06
Bloating	0,18	-0,16	**-0,66**	0,15
Nausea	0,00	0,05	**-0,65**	0,05
Abdominal bloating	0,29	-0,26	**-0,62**	0,50
Early satiety	-0,06	0,09	**-0,57**	0,17
Burping or regurgitation	0,16	0,38	**-0,55**	0,10
Epigastric pain	0,24	0,17	**-0,52**	-0,08
Heartburn	0,27	0,38	**-0,51**	-0,03
Food gets stuck	0,01	0,15	**-0,42**	0,13
Swallowing of air	0,02	0,23	**-0,33**	0,06
Hard or lumpy stools	0,24	0,02	-0,02	**0,67**
A feeling of incomplete emptying	0,33	-0,01	-0,07	**0,61**
Incomplete evacuation	0,16	0,10	-0,03	**0,60**
Straining	0,18	0,10	-0,19	**0,57**
Manual help to finish evacuation	-0,02	0,19	0,01	**0,57**
<three bowel movements a week	-0,05	-0,10	-0,10	**0,33**
Slemish residue	0,31	0,15	-0,05	**0,32**
Epigastric discomfort	0,01	-0,17	-0,05	0,13

Bold figures indicate values > cut off 0.30.

*Crohnbach's alpha *was used to test the internal consistency of the relevant questions from the three main predefined domains (FH, FD, and IBS). All questions were dichotomized into nominal yes/no except no 34, which was used as ordinal data (0 = small amount, 1 = large amount). A high alpha coefficient suggests that the items within a domain measure the same construct, which supports the hypothesis of the internal consistency [18]. A minimum correlation of 0.70 is usually considered necessary, and alpha coefficient values above 0.90 are optimal to allow for individual comparisons [19,20]

## Ethical approval

The study was approved by Forskningsetikkommitté Syd (South ethical committee) Karolinska Institutet. Dnr 394/01.

## Results

### Translation

The words in the final version of the Swedish questionnaire must cover the same meaning as the words n the English questionnaire. English words as *abdomen*, *stomach*, and *pain *can be accurately translated into Swedish in various ways. We compared the back-translation with the original English version and found a few variations in choice of words or terminology, understandable in either language. However, the final wording of the Swedish questionnaire did not change the initial meanings of the questions.

### Feasibility

Forty-one patients answered questions about the feasibility of the questionnaire as described above. A majority found the questionnaire easy to fill in (98%) and easy to understand (93%). Seventy-one percent reported that the description of symptoms was correct and 39% thought that correct questions or wordings correlated to their symptoms were missing. Most of the respondents (59%) needed less than 10 minutes to fill in the questionnaire, 37% needed 10-15 minutes and 5% wanted more than 15 minutes. The patients from the GI clinic needed slightly more time than the patients from the general practice.

### Reproducibility

One hundred and one persons (described above) filled in the questionnaire twice within a week. The kappa values were 0.60 (95% CI ± 0.21) for GERS, 0.52 (95% CI ± 0.27) for FD, and 0.47 (95%CI ± 0.25) for IBS.

Kappa values for the key questions (see Additional file 1) were 0.59 (95%CI+0.19) for Q8, 0.67 (95CI+0.15) for Q10, and 0.30 (95%CI +0.19) for Q20.

### Predictability

Predictability was estimated exclusively from the population sample (Popcol study) and not from patients in order to avoid bias from health seeking behaviour.

Three different analyzes were conducted.

1. Comparison between the diagnoses by the printed version of Rome II and the diagnoses made by the clinician (n = 125). The kappa values and overall agreement were 0.26 (95%CI ± 0,17) and 90%for GERS, 0.18 (95%CI ± 0.16) and 85% for FD, and 0.49 (95%CI ± 0.17) and 86% for IBS, all calculated on a prevalence of 8.8% (n = 11), 6.4% (n = 8) and 15.2% (n = 19) for GERS, FD, and IBS respectively.

When we used clinicians' diagnoses as the criterion standard, the positive predictive value of Rome II was10.5% for FH/GERS, 21.1% for FD, and 63.2% for IBS. The negative predictive value was 96.2% for GERS, 90.5% for FD and 81.1% for IBS.

2. The predictability of the digital version of Rome II was compared to the diagnoses made by the clinicians (n = 1101). The Kappa values, and overall agreement were 0.33 (95%CI ± 0.06) and 88% for GERS, 0.21 (95%CI ± 0.06) and 88%for FD, and 0.43 (95%CI ± 0.06) and 84% for IBS. The prevalence of GERS 10.4% (n = 114), of FD 6.5% (n = 71) and of IBS 14.4% (n = 158). The ability to find healthy individuals had an overall agreement in 60% of the cases. The positive and negative predictive values of having or not having the respective diagnoses by means of Rome II with the clinician's diagnosis as criterion standard, were 34.2% and 95.1% for GERS, 33.8% and 92.2% for FD, and 63.3% and 87.1% for IBS.

3. The kappa values and overall agreement between the printed version and the digital version of Rome II (n = 120) were 0.50 (95%CI ± 0.18) and 92% for GERS, 0.64 (95%CI ± 0.18) and 95% for FD, and 0.76, (95%CI ± 0.18) and 95% for IBS.

### Reliability

#### Principal Component Analysis

PCA was applied to all 237 completed paper questionnaires. Analyses with 2-6 factors were applied in the evaluation, all with an eigenvalue >1. The outcome was compared to the supposed logical outcome.

After analysing versions with 2-6 factors we found that the four-factor table fit the data best in the short version (Table 1) and the five factor table in the long version (Table 2).

**Table 2:** The rotated (long version) PCA of all symptom symptoms listed in the Rome II Modular Questionnaire with five descriptively labelled factors in descending eigenvalues.

Eigenvalue	6.40	4.03	2.47	2.20	2.14
					
Factor label	GERD	IBS/Constip	IBS Misc	Dyspepsia	**Diarrhoea/incont**.
A lump in your throat	**0,75**	-0,08	0,09	0,03	-0,44
Difficult or painful swallowing	**0,65**	-0,01	0,03	-0,12	-0,34
Food regurgitates	**0,60**	0,11	-0,19	-0,19	-0,31
Nausea or vomiting	**0,58**	-0,03	-0,06	0,18	0,07
Regurgitation stops when food turns acid	**0,51**	-0,10	-0,02	-0,04	-0,09
Chest pain	**0,49**	-0,08	0,31	-0,36	-0,30
Food gets stuck	**0,49**	0,04	0,14	-0,57	0,06
Heartburn	**0,49**	-0,14	0,05	-0,44	-0,26
Difficult swallowing	**0,45**	-0,08	0,12	-0,07	-0,30
Epigastric pain	**0,44**	-0,23	0,27	0,05	-0,35
Epigastric discomfort	**0,41**	-0,17	-0,05	-0,73	0,00
Nausea	**0,37**	-0,08	0,15	-0,30	-0,06
Bloating	**0,36**	-0,29	0,07	-0,65	-0,09
Early satiety	**0,34**	-0,02	-0,03	-0,39	0,00
Burp or regurgitation	**0,33**	-0,22	0,13	-0,37	-0,02
Change in stool consistency	0,15	**-0,80**	-0,01	0,08	-0,16
Lower abdominal pain or discomfort (PoD)	0,20	**-0,75**	-0,01	-0,19	-0,07
Change in stool frequency	0,15	**-0,73**	0,03	-0,02	-0,23
PoD diminishes after bowel movements	0,17	**-0,72**	0,16	0,00	0,03
Persistent abdominal pain	-0,02	**-0,53**	-0,01	-0,06	-0,27
Incomplete emptying	-0,12	**-0,52**	-0,07	-0,37	0,06
Anal pain	-0,09	**-0,49**	-0,04	-0,34	-0,06
Difficulties in anal relaxation	-0,17	**-0,39**	0,00	-0,19	0,22
Straining 3/4 of times	-0,10	**-0,38**	-0,06	-0,08	0,01
Hard or lumpy stools	0,09	0,02	**0,68**	-0,09	0,10
Abdominal bloating	0,01	0,05	**0,65**	-0,23	0,04
<three bowel movements a week	0,13	-0,03	**0,64**	-0,06	-0,12
Slemish residue	0,01	-0,04	**0,61**	0,01	-0,22
A feeling of incomplete emptying	0,01	0,01	**0,58**	-0,09	0,14
Loose stools	0,09	0,02	**0,50**	0,06	-0,38
Straining	0,05	-0,07	**0,45**	0,20	-0,30
>three bowel movements a day	0,10	-0,06	**0,42**	0,11	-0,49
Amount of leaking	-0,09	-0,08	-0,14	**-0,36**	-0,74
Bile cholic	-0,03	-0,07	0,09	**-0,36**	-0,27
Anal incontinence	-0,04	-0,09	-0,15	**-0,31**	-0,75
Loose stools 3/4 of times	-0,01	-0,23	0,05	0,09	**-0,52**
Urgency	0,02	0,17	0,17	-0,04	**-0,36**
Swallowing of air	0,25	-0,15	0,15	-0,10	-0,03
Incomplete evacuation	0,00	0,07	0,12	0,12	0,14
Manual help to finish evacuation	-0,01	0,17	0,17	-0,02	0,03
Frequent episodens of vomiting	0,22	0,00	0,21	0,18	-0,19

Bold figures indicate values > cut off 0.30.

#### Chronbach's alpha

For the Cronbach's alpha coefficient, the questions regarding plain symptoms belonging to each domain were introduced, while questions on symptom negations, frequency and non-symptom questions related to a symptom question were left out.

The Cronbach's alpha coefficient for GERS was 0.75 with a span per item of 0.71 to 0.76. For FD the figures were 0.68 and 0.54 to 0.70 (the lowest figure 0.54 for epigastric pain or discomfort). For IBS the figures were 0.61 and 0.56 to 0.66.

## Discussion

Overall, we found that the Swedish version of the Rome II questionnaire is of doubtful accuracy for both research and clinical use. The digital and the paper version gave corresponding results.

An instrument translated into another language must be considered as a new instrument. The questions in the new language must be easy to understand but also expressed in a way that eliminates ambiguity. For example words as "often" or "rarely" must be followed by an explanation of what these words mean in the actual context.

A board of physicians with a special interest in gastroenterology constructed the Rome II questionnaire. It is a result of an ongoing process with structured evaluation of the literature and experts' consensus discussions derived from the Delphi method [21]. However, to quote the Rome II book: "Since there are no observed defects, we only know of these disorders through the words of our patients", and: "Validation studies are difficult and rare". The first statement has really been shown to be true [7].

A drawback in the study might be the possible influence by organic disease on the diagnosis "functional". However 756 participants in the Popcol study had a colonoscopy that included routine biopsy staining from specimens obtained at five levels (four in the colon and one in the distal ileum). The answers to the Rome II questionnaire indicated that 106 of these had IBS. Only six (5.9%) had an organic explanation for their symptoms: one had Crohn's disease, two had lymphocytic colitis, two had collagen colitis, and one had celiac disease. (The Popcol study, Dr Lars Kjellström, personal communication). In another Swedish population based upper endoscopy study 38% reported dyspepsia, but only 4.1% had a peptic ulcer. Only every second of these (54%) had dyspeptic symptoms [8]. Of those with GERS every forth (24 5%) had visible esophagitis [22]. It is common and according to the literature in epidemiological studies relevant to assume that the proportion of individuals with an organic disease is negligible, except for GERS of whom a substantial proportion seems to have an organic cause for their symptoms.

We found the translation well corresponding to the original version and the questionnaire easy to fill in and understand. There was, however, a slight difference between patients in general practice and those in the specialist GI clinics. A few patients from general practice judged that the questionnaire did not describe their symptoms correctly, perhaps because they were less familiar with the terminology than patients from the GI clinic who probably had more practice discussing their symptoms with health care professionals.

The outcome of the reproducibility test, performed within a week after the questionnaire was first administered, was deemed as "moderate", with the best result for GERS. We consider this acceptable in view of the outcome of the factor analysis, the conditioning in the codes for the symptom domains, the relatively few participants, and also the known natural history of change of symptoms over short time, [12,23].

The size of the samples, used in groups A, B, and C might be questioned. There is, however, no possibility to conduct a proper power analysis. We have used sample sizes that are in agreement with the sample sizes used in many other studies in the field of validation of questionnaires [24]. Published recommendations for PCA state that the number of observations should be about 10 times the number of items. For the long PCA we had 6.1 and for the short one 8.1, which is deemed to be acceptable, especially as in many published studies analyses were performed with much lower ratios.

Agreement between the diagnoses made, using the two versions of the questionnaire and by the clinician was fair for GERS and FD but moderate for IBS, This relative inconsistency in agreement creates major doubts about the applicability of the questionnaire at various levels in clinical practice and also to research purposes. However, the inconsistency in the results might also be due to unskilled doctors. We find this unlikely, as all doctors involved in the study were very experienced gastroenterologists, working at one of the most reputable GI centres in Sweden. Moreover, during the study, repeated consensus meetings were held at regular intervals. These meetings focused on the main functional gastrointestinal diagnoses reported in the study. A more probable cause is that the doctors consider the nuances of what a patient says and the eventual predominance of certain symptoms when making a diagnosis. Such interpretation is not possible with the questionnaire and is always problematic when communication is not face-to-face.

Another explanation for the inconsistency might be that the questionnaire is insufficient regarding the symptom questions per se. One reason of this view is the construction of the codes for FH/GERS and FD, as both cannot be diagnosed at the same time. This is known to be clinically irrelevant [25] and also shown to be a misnomer when compared to the outcome of the PCA.

A computerized investigation substantially eases the logistic [26] of recording symptoms; therefore it was of great value that we could show the positive concordance between the two versions. We searched for both in the literature and among experts but could not find any publication that compared the use of a digital and a paper version of any questionnaire in either clinical practice or research.

We have not found any publication on reproducibility of the Rome II questionnaire. However, Aro et al analysed reproducibility of a similar questionnaire (Abdominal Symptom Questionnaire, ASQ) and reported kappa values, higher than ours: for GERS 0.72, for dyspepsia 0.72 and for and IBS 0.78 [27]. This might point out the more complex and therefore less valid structure of the Rome II Patient Modified Formula Questionnaire.

We have searched but not found any publication that presents statistical data concerning the predictability of medical history data.

The best corresponding values were achieved for IBS. The PCA identified the expected symptom domains reasonably well, and together with the outcome of the Chronbach's alpha analysis we found the internal consistency of the digital and the paper version acceptable.

To the best of our knowledge, the Rome II questionnaire as such has never been thoroughly validated. However, diagnoses made using the Rome II criteria have been judged and compared to diagnoses, made in clinical practice. A Russian study [28] found that the questionnaire frequently ended up in multiple diagnoses and therefore was only modestly helpful when applied to consulting patients.

Two Norwegian studies have compared the diagnoses based on the Rome II criteria to diagnoses made by doctors in primary care [26,29]. Both used a questionnaire, based on the Rome II criteria, translated into Norwegian, that included additional questions about duration of symptoms, presence of alarm symptoms, and stress related symptoms. Farup et al [29] studied patients with upper gastrointestinal complaints at the actual visit to a general practitioner and concluded that the Rome II criteria should be used only as an aid to improve the precision of the classification of functional disorders. Vandvik et al [26] concluded that diagnosing IBS on the basis of the Rome II criteria did not correspond to diagnosing IBS patients in general practice. The poor agreement between diagnoses based on the Rome II and practitioners' diagnoses might depend on overly restrictive criteria in Rome II.

Thus, despite all efforts to create diagnostic aids for functional gastrointestinal disorders, it appears that neither general practitioners nor specialists benefit from using them [26,29,30].

While this investigation was underway, a new version, Rome III, was introduced [31]. The main difference between the two versions is the criteria for the length of symptoms. Rome II states that symptoms must be present during at least 3 weeks (at least one day in each week) in the last 3 months, while Rome III states that symptoms must be present during the last three months and includes further questions about frequency (from less than one day a month to every day).

Criteria for FH and IBS are almost identical in the two versions. However, Rome III asks about more detailed symptoms with regard to FD (bothersome postprandial fullness, early satiation, epigastric pain and epigastric burning) while Rome II only asks about "persistent or recurrent symptoms" (pain or discomfort in the upper abdomen).

A few studies that compare results of Rome II and Rome III have been published with conflicting results. The likelihood of identifying patients with IBS was similar in a study by Wang et al. with 3014 patients in an outpatient gastrointestinal clinic [32]. The detection rate was 18.5% with Rome II and 15.9% with Rome III. Sperber at al reported a significant difference between the two versions in diagnosing IBS: 2.9% prevalence when Rome II was used and 11.4% prevalence when Rome III was used [33].

## Conclusion

We found that the Swedish version of the Rome II questionnaire corresponded well to the original English text. The questionnaire was well accepted, easy to use and understand, and covered essential symptom domains with acceptable reproducibility. The ability to predict a diagnosis by the printed and the digital versions seems to be comparable especially for IBS. However, the questionnaire's low ability to predict diagnoses made by experienced clinicians raises doubts about its predictability and indicates the need to further improve the tool. The findings of this study are probably also valid for FH/GERS and IBS in the new version, Rome III. It is clear that future Rome criteria should be validated in large-scale investigations.

## Competing interests

The authors declare that they have no competing interests.

## Authors' contributions

HM planned and fulfilled the work with the collected material, and drafted the manuscript.

LK was responsible for the logistics in the main colonoscopy study (Popcol).

HN was the mentor of LK and participated in the face validity process of the translation. LK also participated in the writing of the manuscript.

LA had the comprehensive responsibility for the main colonoscopy study (Popcol), performed the statistical analyses in our study and worked close to HM to finalize the manuscript.

All authors have read and approved the manuscript.

## Supplementary Material

Additional file 1 Rome II Modular questionnaire, Respondent Form in English. Click here for file

Additional file 2 Rome II Modular Questionnaire: Respondent Form, translated into Swedish. Click here for file
