# Supplementary material for: Doubtful outcome of the validation of the Rome II questionnaire: validation of a symptom based diagnostic tool
Source: Health Qual Life Outcomes. 2009 Dec 29;7:106. doi: 10.1186/1477-7525-7-106 (PMC2806864; doi:10.1186/1477-7525-7-106)
Supplement: Additional file 1 — Rome II Modular questionnaire, Respondent Form in English. [file 1477-7525-7-106-S1.DOC]

# Additional file 1

# Rome II Modular Questionnaire: Respondent Form

Question Answer

# Esophageal Symptoms

1. In the last 3 months, did you often* 🞐_0_ No or rarely → skip to

get the feeling of a lump in your question 3

throat when you were *not* swallowing? 🞐_1_ Yes 

1. When you are eating or drinking, is 🞐_0_ No or rarely

it difficult to swallow, or does it hurt to 🞐_1_ Yes

swallow?

1. In the last 3 months, did you often* 🞐_0_ No or rarely → skip to

bring up food, chew it again, and either question 6

spit it out or re-swallow it? 🞐_1_ Yes 

1. At these times, did you vomit or feel 🞐_0_ No or rarely

sick to your stomach? 🞐_1_ Yes 

1. Do you stop bringing up food when 🞐_0_ No or rarely

the food turns sour (acidic)? 🞐_1_ Yes 

______________________________________________________________________________

1. In the last 3 months, did you often* 🞐_0_ No or rarely → skip to

have pain in the middle of your chest   question 8

(that is not due to angina or a heart attack)? 🞐_1_ Yes

1. Did this chest pain occur when it felt 🞐_0_ No or rarely

like food got stuck going down? 🞐_1_ Yes 

1. In the last 3 months, did you often* 🞐_0_ No or rarely

have heartburn, a burning pain or 🞐_1_ Yes 

discomfort in your chest ((that is not due to

angina or a heart attack)?

1. In the last 3 months, did you often* 🞐_0_ No or rarely

have difficulty after swallowing (solid 🞐_1_ Yes 

or liquids sticking in your chest, or

passing down normally)?

________________________________________________________________________________

- *Often* means that the symptoms were present during at least 3 weeks (at least one day in each week) in the last 3 months.

Question Answer

# Gastroduodenal symptoms

1. In the last 3 months, did you often* 🞐_0_ No or rarely → skip to

have discomfort or pain centered in question 15

your upper abdomen (above your 🞐_1_ Yes

belly button, or in the pit of your

stomach?

1. Check your best description of this 🞐_1_ *pain* in your skip to

symptom or the one that bothers abdomen or question 13

your most stomach

🞐_2_ *discomfort* (that is

not painful) in your

upper abdomen or

stomach

1. If you have discomfort, which of 🞐_1_ nausea

the following describe your discomfort? 🞐_2_ bloating (a sensation

(check all that apply) of upper abdominal

swelling)

🞐_3_ feeling full after eating

very little

🞐_4_ none of the above

1. Does your upper abdominal 🞐_0_ No or rarely

discomfort or pain usually get better or stop 🞐_1_ Yes

after you have a bowel movement?

14a. When the upper abdominal 🞐_0_ No or rarely

discomfort or pain starts, do you usually 🞐_1_ Yes

have a change in your usual number

of bowel movements (either more or

fewer)?

14b. When the upper abdominal discomfort 🞐_0_ No or rarely

or pain starts, do you usually have either 🞐_1_ Yes

softer or harder stools than usual?

15, In the last 3 months, did you often* 🞐_0_ No or rarely → skip to

burp or belch? question 17

🞐_1_ Yes

16. Did you swallow air to help you belch? 🞐_0_ No or rarely

🞐_1_ Yes

__________________________________________________________________________________

- *Often* means that the symptoms were present during at least 3 weeks (at least one day in each week) in the last 3 months.

Question Answer

1. In the last 3 months, did you have 🞐_0_ No or rarely → skip to

frequent episodes of vomiting (on at question 20

least 3 separate days in each week)? 🞐_1_ Yes

1. During these episodes, did you make 🞐_0_ No or rarely → skip to

yourself vomit? question 20

🞐_1_ Yes

1. Were you vomiting because of a 🞐_0_ No or rarely

medication you were taking or 🞐_1_ Yes

another medical condition that you had?

______________________________________________________________________________

# Bowel Symptoms

1. In the last 3 months, did you *often** 🞐_0_ No or rarely skip to

have discomfort or pain in your 🞐_1_ Yes question 24

abdomen?

1. Does your discomfort or pain get 🞐_0_ No or rarely

better or stop after you have a 🞐_1_ Yes

bowel movement?

1. When the discomfort or pain starts, 🞐_0_ No or rarely

do you have a change in your usual 🞐_1_ Yes

number of bowel movements (either more

or fewer9?

1. When the discomfort or pain starts, 🞐_0_ No or rarely

do you have either softer or harder 🞐_1_ Yes

stools than usual?

*Often* means that the symptoms were present during at least 3 weeks (at least one day in each week) in the last 3 months

1. Have you had any of the following 🞐_1_ Fewer than three

symptoms at least one forth (1/4) bowel movements a

of the time (occasions or days) in the week(0-2)

last 3 months?(check all that apply). 🞐_2_ More than three

bowel movements a

day (4 or more)

🞐_3_ Hard or lumpy stools

🞐_4_ loose, mushy or

watery stools

🞐_5_ Straining during a

bowel movement

🞐_6_ Having to rush to

the toilet to have a

bowel movement

🞐_7_ Feeling of incomplete

emptying after

a bowel movement

🞐_8_ Passing mucus (slime)

during a bowel movement

🞐_9_ Abdominal fullness,

bloating or swelling

🞐_10_ A sensation that the stool

cannot be passed (i.e. blocked)

when having a bowel movement

🞐_11_ A need to press on or around

your bottom or vagina to try

to remove stool in order to

complete the bowel movement.

1. In the last 3 months, did you have 🞐_0_ No

loose, mushy or watery stools, 🞐_1_ Yes

during more than three quarters (3/4)

of your bowel movements?

________________________________________________________________________________

# Abdominal Pain Symptoms

26. In the last 6 months, did you have 🞐_0_ No Skip to

pain in your abdomen all the time question 28

(continuously) or most of the time 🞐_1_ Yes

(nearly continuously)? *(if you are*

*female, this should not be related to*

*your menstrual cycle or period*)

1. Has this pain limited or restricted 🞐_0_ No or rarely

your ability to work or go to 🞐_1_ Yes

social events?

*Often* means that the symptoms were present during at least 3 weeks (at least one day in each week) in the last 3 months

# Question Answer

# ________________________________________________________________________________

# Biliary Symptoms

1. In the last year, did you have any 🞐_0_ No or rarely Skip to

severe steady pain in the middle or question 33

right side of your upper abdomen? 🞐_1_ Yes

1. Did the pain last 30 minutes or more? 🞐_0_ No or rarely

🞐_1_ Yes

30. Did the pain keep you from your usual daily 🞐_0_ No or rarely

activities, or cause you to see a doctor? 🞐_1_ Yes

1. Have you had your gallbladder removed? 🞐_0_ No Skip to

question 33

🞐_1_ Yes

1. Did you have any severe or steady 🞐_0_ No or rarely

pain in the middle or right side of 🞐_1_ Yes

your abdomen since your gallbladder

was removed?

. __________________________________________________________________________________

# Anorectal symptoms

1. In the last year, when you had constipation 🞐_0_ No Skip to

or diarrhoea, did you accidentally leak question 35

or pass stool for more than one occasion 🞐_1_ Yes

in a month?

1. How much stool did you accidentally 🞐_1_ A small amount (it

lose. Would you say…… stains underwear)

🞐_2_ A moderate or large

amount (2 teaspoons

or more.

1. In the last year, did you have more than 🞐_0_ No Skip to

one episode of aching pain or question 38

pressure in the anal canal or rectum? 🞐_1_ Yes

.

1. Did this pain occur frequently or 🞐_0_ No

continuously in the last 3 months? 🞐_1_ Yes

*Often* means that the symptoms were present during at least 3 weeks (at least one day in each week) in the last 3 months

Question Answer

__________________________________________________________________________________

1. Which of the following 2 statements 🞐_1_ Lasts from seconds to

better describes the aching, pain, or minutes and disappears

pressure that you had in the anal canal completely.

or rectum? 🞐_2_ Lasts more than 20

minutes and up to several days

or longer.

1. In the last 3 months, when you were 🞐_1_ Feel as if you had to

having bowel movements, did you… strain to pass your….

(check all that apply) stool at least one quarter

of the time

🞐_2_ Feel as if you were unable

to empty the rectum at least

one quarter of the time

🞐_3_ Have difficulty relaxing

or letting go to allow the

stool to come out at least

one quarter of the time

🞐_4_ None of the above

End of Questionnaire
