# Supplementary material for: Doubtful outcome of the validation of the Rome II questionnaire: validation of a symptom based diagnostic tool
Source: Health Qual Life Outcomes. 2009 Dec 29;7:106. doi: 10.1186/1477-7525-7-106 (PMC2806864; doi:10.1186/1477-7525-7-106)
Supplement: Additional file 2 — Rome II Modular Questionnaire: Respondent Form, translated into Swedish. [file 1477-7525-7-106-S2.DOC]

# Additional File 2

# Rome II Patientanpassat frågeformulär

Fråga Svar

# Symtom från matstrupen

1. Under de senaste 3 månaderna, 🞐_0_ Nej eller sällan gå till

har du ofta* haft en känsla av fråga 3

”klump i halsen” 🞐_1_ Ja

trots att du inte svalde?  

1. När du äter eller dricker, 🞐_0_ Aldrig eller sällan

är det svårt att svälja eller gör det 🞐_1_ Ja

ont när du sväljer?

1. Under de senaste 3 månaderna, har det 🞐_0_ Nej eller sällan → gå till

ofta* hänt att maten kommer upp igen fråga 6

och att du är tvungen att spotta ut den 🞐_1_ Ja 

eller svälja den igen?

1. Hände detta vid de tillfällen då du 🞐_0_ Nej eller sällan

kände dig illamående eller hade kräkts? 🞐_1_ ja 

1. Slutade du få upp maten när den 🞐_0_ Nej eller sällan

smakade surt? 🞐_1_ Ja 

______________________________________________________________________________

1. Under de senaste 3 månaderna, har du ofta* 🞐_0_ Nej eller sällan gå till

haft en smärta mitt i bröstet   fråga 8

(som inte berodde på kärlkramp 🞐_1_ Ja

eller hjärtbesvär)?

1. Uppträdde denna bröstsmärta när det kändes 🞐_0_ Nej eller sällan

som om maten fastnade på väg ned? 🞐_1_ Ja 

1. Under de senaste 3 månaderna, har du ofta* 🞐_0_ Nej eller sällan

haft halsbränna eller bröstbränna 🞐_1_ Ja 

(obehag i bröstet som inte berodde på kärlkramp

eller hjärtattack)?

1. Under de senaste 3 månaderna, har du ofta* 🞐_0_ Nej eller sällan

haft besvär efter att du har svalt (fast 🞐_1_ Ja 

eller flytande föda fastnade i bröstet eller

passerade inte som vanligt)?

________________________________________________________________________________

- *Ofta* betyder symtom under åtminstone 3 veckor (åtminstone en dag varje vecka) under de senaste 3 månaderna

® H.Molinder et al. Family medicine, Stockholm 2002

Fråga Svar

# Symtom från mage och tarm

1. Under de senaste tre månaderna, 🞐_0_ Nej eller sällan → gå till

har du ofta* haft smärta eller obehag fråga 15

i den övre delen av magen (ovanför 🞐_1_ Ja

naveln eller i maggropen)?

1. Markera den bästa beskrivningen 🞐_1_ *Smärta* i övre magen

av dina symtom eller det som besvärar eller buken gå till dig mest fråga 13

🞐_O_*obehag*, (som inte är

smärtsamt) i övre delen av magen

1. Om du har obehag,, vilket eller 🞐_1_ Illamående

vilka av dessa symtom beskriver ditt 🞐_2_ Uppblåsthet (en känsla

obehag? av att övre delen av magen sväller)

(markera alla som passar in) 🞐_3_ Känsla av att vara mätt

trots att du ätit mycket litet.

🞐_4_ Ingenting av ovanstående

1. Brukar ditt obehag eller din smärta 🞐_0_ Nej eller sällan

i övre delen av magen lindras eller försvinna 🞐_1_ Ja

helt efter tarmtömning?

14a. När ditt obehag eller din smärta i övre 🞐_0_ Nej eller sällan

delen av magen börjar, brukar 🞐_1_ Ja

antalet tarmtömningar förändras

(bli antingen fler eller färre)?

14b. När ditt obehag eller din smärta 🞐_0_ Nej eller sällan

i övre delen av magen börjar, brukar du då 🞐_1_ Ja

få lösare eller hårdare avföring än vanligt?

15, Under de senaste 3 månaderna, har du ofta 🞐_0_ Nej eller sällan → gå till

haft rapningar eller uppstötningar? fråga 17

🞐_1_ Ja

16. Har du svalt luft för att lättare kunna rapa? 🞐_0_ Nej eller sällan

🞐_1_ Ja

__________________________________________________________________________________

- *Ofta* betyder symtom under åtminstone 3 veckor (åtminstone en dag varje vecka) under de senaste 3 månaderna

® H.Molinder et al. Family medicine, Stockholm 2002

Fråga Svar

1. Under de senaste 3 månaderna, har du kräkts 🞐_0_ Nej eller sällan → gå till

ofta (åtminstone 3 olika dagar varje fråga 20

vecka)? 🞐_1_ Ja

1. Vid dessa tillfällen, framkallade 🞐_0_ Nej eller sällan → gå till

du själv kräkningarna? fråga 20

🞐_1_ Ja

1. Kräktes du på grund av någon 🞐_0_ Nej eller sällan

medicin du tog eller någon sjukdom 🞐_1_ Ja

du hade?

______________________________________________________________________________

# Tarmsymtom

1. Under de senaste 3 månaderna, har du 🞐_0_ Nej eller sällan → gå till

ofta* haft obehag eller smärta i buken? fråga 24

🞐_1_ Ja

1. Brukar ditt obehag eller din smärta 🞐_0_ Nej eller sällan

lindras eller försvinna helt efter 🞐_1_ Ja

tarmtömning?

22 När ditt obehag eller din smärta i buken 🞐_0_ Nej eller sällan

börjar, brukar antalet tarmtömningar 🞐_1_ Ja

förändras (bli antingen fler eller färre)?

23. När ditt obehag eller din smärta 🞐_0_ Nej eller sällan

i buken börjar, brukar du ha lösare 🞐_1_ Ja

eller hårdare avföring än vanligt?

Se fråga 24 på nästa sida

**Ofta* betyder symtom under åtminstone 3 veckor (åtminstone en dag varje vecka) under de senaste 3 månaderna

® H.Molinder et al. Family medicine, Stockholm 2002

1. Har du haft något av följande 🞐_1_ Färre än tre avföringar

symtom åtminstone en fjärdedel i veckan (2 eller färre).

(1/4) av tiden (avföringstillfällen eller 🞐_2_ Mer än tre avföringar per dag.

dagar) under de senaste 3 månaderna? (4 eller fler).

(markera alla som passar in). 🞐_3_ Hård eller klumpformad avföring.

🞐_4_ Lös, grötig eller vattnig avföring

🞐_5_ ansträngande krystning för att kunna tömma tarmen.

🞐_6_ Varit tvungen att rusa till toaletten

för att tömma tarmen.

🞐_7_ En känsla av ofullständig tömning

efter avföring.

🞐_8_ Att det kommer slem tillsammans med avföringen.

🞐_9_ Svullnad, uppkördhet eller ,

uppblåsthet av buken.

🞐_10_ En känsla av att avföringen

stoppade upp när du skulle ha avföring

🞐_11_ Behöver du trycka i eller runt

ändtarmsöppningen eller slidan med

fingrarna för att få ut avföringen och

avsluta tarmtömningen?

25. Under de senaste 3 månaderna 🞐_0_ Nej

har du haft lös, grötig eller vattnig 🞐_1_ Ja

avföring mer än tre fjärdedelar (3/4)

av dina tarmtömningar?

________________________________________________________________________________

**Symtom på buksmärta**

26. Under de senaste 3 månaderna, 🞐_0_ Nej Gå till

har du haft ont i buken ihållande fråga 28

(konstant) eller i stort sett ihållande 🞐_1_ Ja

(nästan konstant)? *(om du är kvinna,*

*skall detta inte relateras till din*

*menstruation)*

27 Har denna smärta begränsat 🞐_0_ Nej eller sällan

eller hindrat din möjlighet 🞐_1_ Ja

att arbeta eller att deltaga i sociala

aktiviteter?

*Ofta* betyder symtom under åtminstone 3 veckor (åtminstone en dag varje vecka) under de senaste 3 månaderna

® H.Molinder et al. Family medicine, Stockholm

Fråga Svar

________________________________________________________________________________

**Symptom från gallan**

28. Under det senaste året, har du haft 🞐_0_ Nej eller sällan Gå till

någon svår eller ihållande smärta mitt i övre fråga 33

eller högra övre delen av buken? 🞐_1_ Ja

29. Varade smärtan 30 minuter eller mer? 🞐_0_ Nej eller sällan

🞐_1_ Ja

30. Hindrade smärtan dina dagliga aktiviteter 🞐_0_ Nej eller sällan

eller måste du söka läkare? 🞐_1_ Ja

31. Är din gallblåsa bortopererad? 🞐_0_ Nej Gå till

fråga 33

🞐_1_ Ja

32 Har du haft någon svår eller ihållande smärta 🞐_0_ Nej eller sällan

mitt i övre eller högra övre delen av buken 🞐_1_ Ja

efter det att din gallblåsa blev bortopererad?

. __________________________________________________________________________________

# Symptom från ändtarmen

33 Under det senaste året, om du hade förstoppning 🞐_0_ Nej Gå till

eller diarré, ”läckte ”du ibland eller fråga 35

fick du okontrollerad avföring oftare än en gång 🞐_1_ Ja

i månaden?

34 Hur mycket avföring kom okontrollerat? 🞐_1_ Litet (som smutsade dina underkläder)

🞐_2_ En ganska stor eller stor mängd

(som 2 teskedar eller mer)

1. Under det senaste året har du haft mer än 🞐_0_ Nej Gå till

ett tillfälle med smärta eller tryck i fråga 38

ändtarmen eller ändtarmsöppningen? 🞐_1_ Ja

.

36 Har du haft denna smärta ofta* eller 🞐_0_ Nej

ihållande under de senaste 3 månaderna? 🞐_1_ Ja

*Ofta* betyder symtom under åtminstone 3 veckor (åtminstone en dag varje vecka) under de senaste 3 månaderna

® H.Molinder et al. Family medicine, Stockholm 2002

Fråga Svar

______________________________________________________________________________

37 Vilket av följande 2 påståenden beskriver 🞐_1_ varade från sekunder till minuter

bäst smärtan eller trycket du hade i och försvann helt

ändtarmen eller ändtarmsöppningen? 🞐_2_ varade mer än 20 minuter

och upp till flera dagar eller mer

1. Under de senaste 3 månaderna när 🞐_1_ kände det som om du

du hade avföring, tyckte du att du……… måste krysta ansträngt för att tömma

(markera alla som passar in) tarmen åtminstone en fjärdedel

av gångerna

🞐_2_ kände det som om du

inte kunde tömma ändtarmen

åtminstone en fjärdedel av gångerna

🞐_3_ hade svårt att slappna av eller

låta avföringen komma ut

åtminstone en fjärdedel av gångerna

🞐_4_ Ingenting av detta

Slut på frågeformuläret

® H.Molinder et al. Family medicine, Stockholm 2002
